# Global burden and future projections of geriatric gout (1990–2021): a comprehensive analysis and Bayesian Age-Period-Cohort modeling

**DOI:** 10.3389/fpubh.2025.1577265

**Published:** 2025-05-01

**Authors:** Jiqing Wang, Yonghui Zhao, Boya Zhao, Yingang Zhang

**Affiliations:** ^1^Department of Orthopedics, The First Affiliated Hospital of Xi’an Jiaotong University, Xi'an, China; ^2^Center for Reproductive Medicine, The First Affiliated Hospital of Zhengzhou University, Zhengzhou, China

**Keywords:** GBD, gout, BAPC analysis, older adult population, YLDs rate

## Abstract

**Introduction:**

Gout is increasingly recognized as a major chronic condition among older adults, contributing significantly to global disease burden, healthcare costs, and disability.

**Methods:**

This study utilized data from the Global Burden of Disease (GBD) database, covering 204 countries and regions from 1990 to 2021. We evaluated age-standardized incidence rates, prevalence, and Years Lived with Disability (YLDs) of gout among individuals aged 60 years and older. Inequality and frontier analyses were conducted to explore disparities, and Bayesian Age-Period-Cohort (BAPC) models were applied for future trend projections up to 2036.

**Results:**

All indicators—incidence, prevalence, and YLDs—showed a steady increase across the study period, with particularly rapid growth observed in high Socio-demographic Index (SDI) regions and among older women. Although the burden was concentrated in high SDI countries, increasing trends were also evident in low SDI areas. Significant heterogeneity was found among countries with similar SDI levels.

**Discussion:**

The projected rise in gout burden through 2036 underscores the urgent need for targeted public health strategies. Disparities across SDI groups suggest that medical infrastructure, prevention programs, and cultural contexts play critical roles in disease control. Comprehensive interventions are essential to manage this growing challenge, especially among high-risk older populations.

## 1 Introduction

Gout is a common inflammatory arthritis, characterized by hyperuricemia and deposition of monosodium urate crystals in joints and soft tissues, leading to severe pain and functional impairment (1). In recent years, the global prevalence of gout has significantly increased, primarily attributed to population aging, changes in dietary habits, and an increased burden of metabolic diseases (2, 3). Although traditionally considered a disease of middle-aged men, latest evidence shows that its impact on the older adult population (aged ≥60) is particularly significant, affecting quality of life, healthcare expenditure, and management of comorbidities.

With the rapid growth of the older adult population, this group faces a series of unique risk factors for gout, including a high incidence of chronic kidney disease, increased medication use (especially diuretics), changes in body composition, and age-related decline in uric acid excretion capacity (4, 5). Studies have shown that the incidence of gout in postmenopausal women has significantly increased, narrowing the gender gap among the older adults with gout (6). Furthermore, older adults often suffer from multiple chronic conditions such as hypertension, type 2 diabetes, and cardiovascular diseases, which exacerbate hyperuricemia and increase the complexity of gout treatment (7). Therefore, gout among older adults has become a significant public health issue globally, necessitating intervention strategies tailored specifically for the older adults rather than younger patients.

Despite extensive epidemiological studies on gout in the general population, comprehensive assessments of the global burden of gout specifically among older adults have only recently begun (8). Previous analyses often overlooked the specific clinical manifestations and demographic characteristics of patients aged 60 and above (9). Moreover, geographical differences are also crucial in understanding the epidemiology of gout, with diet cultures, healthcare systems, and socioeconomic statuses in different regions greatly influencing the incidence and severity of the disease (10, 11). These factors’ in-depth study holds great importance for developing precise prevention strategies, optimizing resource allocation, and enhancing healthcare system capabilities, especially within the growing burden of gout among older adults.

Additionally, the interaction between population aging and metabolic risk factors underscores the importance of long-term trend forecasting (12). Bayesian Age-Period-Cohort (BAPC) models and other advanced statistical methods can be used to predict disease development trends and assess the potential impacts of current policies and interventions (13, 14). However, currently, systematic applications of these models to analyze future trends in older adult gout are still limited. Addressing this knowledge gap will help determine if existing strategies are sufficient to control the growing burden of gout or if more proactive measures are needed.

Therefore, this study uses the Global Burden of Disease (GBD) database to analyze the incidence, prevalence, and Years Lived with Disability (YLDs) of gout in individuals aged 60 and over from 1990 to 2021, stratified by gender, region, and socio-demographic index (SDI). Additionally, inequality indices (SII) and concentration indices (CI) were used to assess the uneven distribution of gout burden, frontier analysis to evaluate the performance of countries at different SDI levels, and BAPC models to predict disease trends up to 2036. By providing an in-depth analysis of the global burden and future trends of gout among older adults, this study aims to offer scientific evidence for public health policy-making and clinical interventions, especially targeting areas where gout-related morbidity is increasing most notably.

## 2 Method

### 2.1 Study design and data sources

This study adopted a population-based ecological design utilizing data from the Global Burden of Disease (GBD) database spanning 204 countries and regions from 1990 to 2021 (15, 16). The GBD data integrates epidemiological information from multiple sources including population registries hospital records household surveys and published literature through standardized protocols. Population denominators and age structures were obtained from the United Nations Population Division (UNPD) to align with international demographic estimates.

### 2.2 Case definition and inclusion criteria

Older adult gout was defined as gout occurring in individuals aged 60 years or older. Gout-related events (incidence prevalence and years lived with disability [YLDs]) were extracted based on International Classification of Diseases (ICD) codes and mapped to the GBD disease classification. Data included: (1) Incidence: new cases of gout diagnosed within a susceptible population in a specific year; (2) Prevalence: all surviving gout patients (both newly diagnosed and previously diagnosed) in a specific year; (3) YLDs: non-fatal disease burden measured by multiplying prevalence by the disability weight for gout derived from the GBD framework. This study primarily analyzed data for individuals aged 60 years and above.

### 2.3 Socio-demographic index (SDI) classification

To analyze heterogeneity across different socioeconomic backgrounds countries were categorized according to the GBD socio-demographic index (SDI). SDI is a composite indicator that considers income per capita average educational attainment and total fertility rate dividing countries into five SDI levels: low low-middle middle high-middle and high. This classification facilitated comparisons of gout burden and trends across varying stages of development.

### 2.4 Statistical analysis

#### 2.4.1 Age standardization

All incidence prevalence and YLD estimates were age-standardized based on the GBD 2021 reference population to ensure comparability across different time points and regions.

#### 2.4.2 Joinpoint regression analysis

Joinpoint regression analysis (Joinpoint Regression Program version 5.1.0.0) was used to identify significant changes in the growth rates of gout incidence prevalence and YLDs. Annual percentage change (APC) and estimated annual percentage change (EAPC) were calculated to quantify overall trends with 95% confidence intervals (CIs) reported. Permutation tests assessed the number of joinpoints using *p* < 0.05 as the significance level.

#### 2.4.3 Decomposition analysis

A decomposition framework was applied to break down the total change in incidence prevalence and YLDs into three components (17): (1) Population Growth – impact of increased total population size; (2) Population Aging – effect of shifts toward an older age structure; and (3) Epidemiological Change – alterations in underlying risk factors (e.g., lifestyle metabolic risk factors). Contributions from these factors were calculated for each country and region between 1990 and 2021.

#### 2.4.4 Inequality measurements

To assess the distributional inequality of gout YLDs across different SDI levels the Slope Index of Inequality (SII) and Concentration Index (CI) were computed. SII represents the absolute difference in YLD rates between the highest and lowest SDI countries while CI reflects the distribution of gout YLDs across different SDI countries based on concentration curves. Negative values indicate higher gout burden in high SDI countries whereas positive values suggest higher burden in low SDI countries.

#### 2.4.5 Frontier analysis

Frontier analysis compared actual YLDs of countries against the “best achievable YLD level” based on SDI expectations. Countries above the frontier line are considered to have higher-than-expected gout YLDs indicating poor control of gout burden.

#### 2.4.6 Bayesian Age-Period-Cohort (BAPC) predictive analysis

We projected the trends in gout burden (YLDs, incidence, and prevalence) from 2022 to 2036 using a Bayesian Age-Period-Cohort (BAPC) model based on the Global Burden of Disease (GBD) 2021 data. Observed data from 1990 to 2021, including both disease metrics and population size stratified by age, sex, and location, were used as input.

The modeling was conducted using the BAPC package in R with Integrated Nested Laplace Approximation (INLA). A 5-year interval structure (gf = 5) and second-order difference priors (secondDiff = TRUE) were specified to ensure continuity across age, period, and cohort dimensions. Age-standardized rates were computed using the GBD global standard population and scaled per 100,000 population.

### 2.5 Software and ethical considerations

Data cleaning descriptive analysis and visualization were primarily conducted using R software (version 4.4.2). As the GBD database comprises publicly available de-identified data this study did not require Institutional Review Board (IRB) approval or informed consent from participants. All analyses adhered to the STROBE (Strengthening the Reporting of Observational Studies in Epidemiology) guidelines complying with GBD research data sharing and ethical agreements.

## 3 Results

### 3.1 Global burden of gout among older adults (1990–2021)

**Figure 1:**
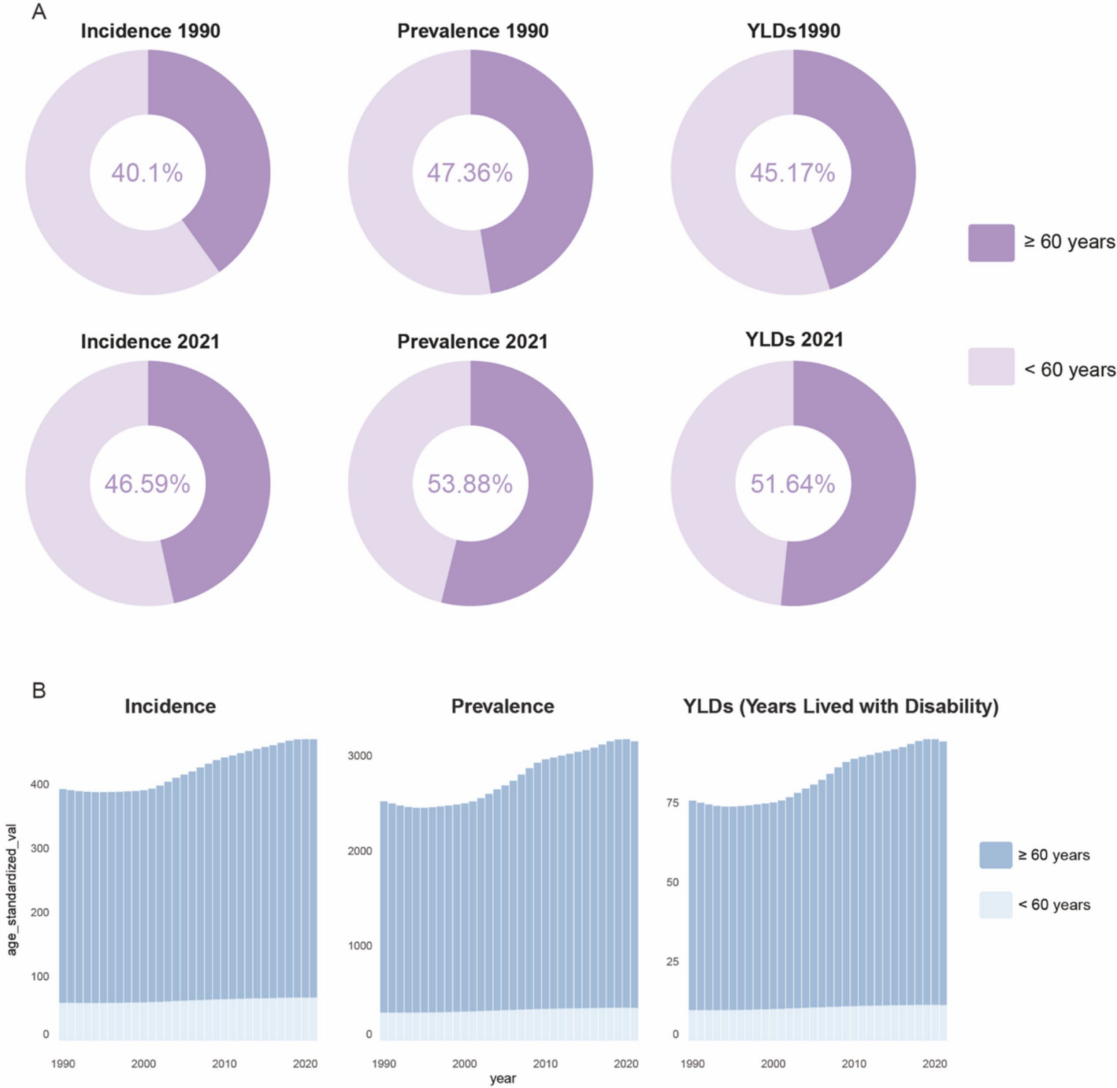
Global proportions of incident cases, prevalent cases, and YLDs numbers for older adult gout **(A)**, and age-standardized trends over time **(B)**. YLDs, years lived with disability.

**Figure 2:**
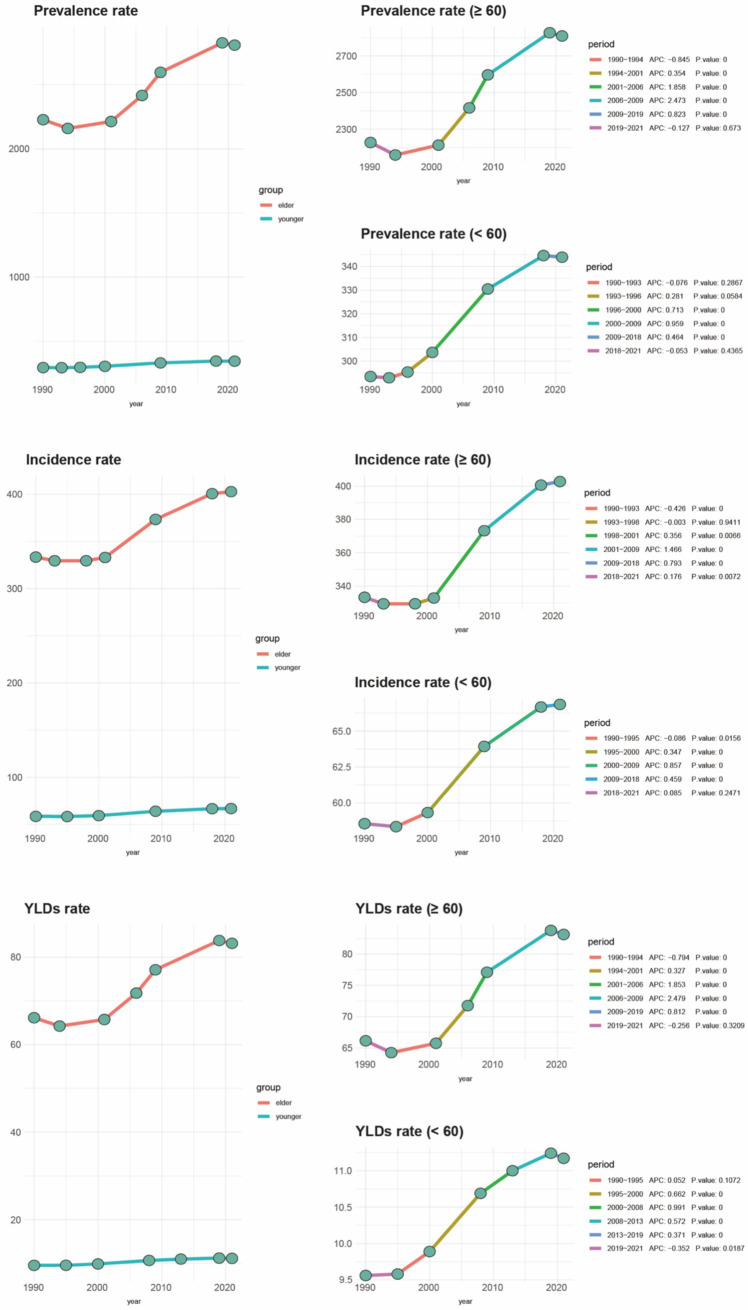
Joinpoint regression analysis of older adult gout compared to other age groups with incidence from 1990 to 2019. OA, osteoarthritis; SDI, socio-demographic index; YLDs, years lived with disability.

Based on global data covering 204 countries and regions (Table 1), from 1990 to 2021, there has been a significant upward trend in age-standardized incidence rates, prevalence, and Years Lived with Disability (YLDs) for gout among individuals aged 60 and over. As shown in Figure 1, the proportion of the gout burden among the older adults has significantly increased: the incidence rate rose from 40.1 to 46.59%, prevalence from 47.36 to 53.88%, and YLDs from 45.17 to 51.64%. Additionally, joinpoint regression analysis (Figure 2) indicates that since around 2000, the growth rates of incidence and YLDs among those aged 60 and over have accelerated and surpassed those under 60.

**Table 1:** The incidence, prevalence, and YLDs, as well as the age-standardized YLDs rates attributable to senile gout in 2021, along with the Estimated Annual Percentage Change globally and across.

	Number of Incident cases (95%UIs)	Age-standardized rate of incidence per 100,000 (95%UIs)	Estimated Annual Percentage Change of incidence per 100,000 from 1990 to 2021 (95%CI)	Number of prevalent cases (95%UIs)	Age-standardized rate of prevalence per 100,000 (95%UIs)	Estimated Annual Percentage Change of prevalence per 100,000 from 1990 to 2021 (95%CI)	Number of YLDs (95%UIs)	Age-standardized rate of YLDs per 100,000 (95%UIs)	Estimated Annual Percentage Change of YLDs per 100,000 from 1990 to 2021 (95%CI)
**Global**	**4380070.51 (2681351.63–6731854.55)**	**401.75 (245.94–617.46)**	**0.85% (0.77–0.93)**	**30425999.41 (20377437.54–43106788.54)**	**2790.72 (1869.05–3953.81)**	**1.12% (1.02–1.23)**	**902502.82 (538446.66–1396209.36)**	**82.78 (49.39–128.06)**	**1.10% (0.99–1.21)**
Male	2976070.19 (1814970.08–4573278.70)	589.56 (359.55–905.97)	0.80% (0.72–0.88)	21281995.62 (14290131.53–30074647.96)	4215.98 (2830.89–5957.81)	1.10% (0.99–1.22)	635015.86 (379610.18–982386.96)	125.8 (75.20–194.61)	1.08% (0.97–1.20)
Female	1404000.32 (866358.39–2164650.01)	239.81 (147.98–369.73)	0.82% (0.73–0.92)	9144003.79 (6038822.98–13075524.53)	1561.84 (1031.46–2233.36)	1.04% (0.92–1.15)	267486.96 (158446.95–414832.87)	45.69 (27.06–70.86)	1.00% (0.89–1.12)
High SDI	1229780.01 (740785.37–1926379.03)	446.95 (269.64–699.43)	1.12% (1.00–1.24)	11554163.84 (7988390.78–15965045.01)	4143.01 (2864.90–5711.16)	1.79% (1.61–1.97)	339240.24 (210875.64–519523.59)	122.46 (76.14–187.42)	1.74% (1.57–1.92)
High-middle SDI	1089801.49 (665683.72–1680406.40)	423.79 (258.89–653.16)	1.04% (0.96–1.12)	6947716.95 (4539663.31–10004245.85)	2699.41 (1763.51–3886.89)	1.11% (1.03–1.19)	207758.08 (121813.11–327763.38)	80.76 (47.34–127.43)	1.11% (1.02–1.19)
Middle SDI	1339379.74 (822797.81–2034369.92)	406.88 (250.12–618.24)	0.84% (0.75–0.93)	7842309.95 (5089650.27–11393571.11)	2388.21 (1550.82–3471.42)	0.95% (0.85–1.05)	234634.22 (136855.79–368320.73)	71.24 (41.59–111.85)	0.93% (0.83–1.03)
Low-middle SDI	539406.51 (332358.68–817811.51)	320.45 (197.37–487.29)	0.30% (0.29–0.31)	3061603.64 (1972130.52–4454838.92)	1827.13 (1178.97–2659.07)	0.34% (0.33–0.35)	90573.37 (52289.45–143729.67)	53.77 (31.13–85.28)	0.34% (0.33–0.35)
Low SDI	178922.81 (110922.78–272,383)	326.05 (202.01–498.01)	0.18% (0.17–0.19)	1003012.82 (646181.30–1460050.26)	1842.65 (1189.40–2683.97)	0.20% (0.19–0.21)	29786.34 (17037.78–46828.30)	54.27 (31.17–85.28)	0.21% (0.20–0.23)
**Central Europe, Eastern Europe, and Central Asia**	**268721.89 (165739.00–409012.85)**	**304.76 (187.73–464.19)**	**0.49% (0.48–0.50)**	**1531533.22 (987628.83–2228117.97)**	**1737.16 (1120.89–2525.67)**	**0.56% (0.55–0.58)**	**45247.12 (26229.67–71993.48)**	**51.34 (29.78–81.60)**	**0.56% (0.55–0.57)**
Central Asia	30059.04 (18409.62–46295.04)	314.03 (192.24–485.69)	0.56% (0.55–0.57)	176207.39 (113132.48–255584.94)	1864.05 (1200.41–2707.94)	0.66% (0.65–0.67)	5312.24 (3026.21–8553.28)	55.82 (31.95–89.54)	0.65% (0.64–0.66)
Central Europe	82805.77 (50917–126702.97)	272.31 (167.36–415.92)	0.45% (0.43–0.46)	473177.95 (305290.92–686931.92)	1548.67 (997.90–2247.03)	0.52% (0.50–0.53)	13956.09 (8069.11–22209.65)	45.84 (26.47–72.99)	0.52% (0.50–0.53)
Eastern Europe	155857.07 (96219.04–236492.36)	323.33 (199.18–491.49)	0.54% (0.53–0.56)	882147.87 (567539.12–1281927.29)	1832.11 (1180.33–2659.08)	0.61% (0.60–0.63)	25978.78 (15026.83–41224.96)	53.95 (31.29–85.41)	0.61% (0.60–0.63)
**High-income**	**1223656.37 (735330.85–1924562.16)**	**429.08 (258.37–674.41)**	**1.04% (0.92–1.16)**	**12037259.80 (8289209.41–16650557.57)**	**4154.70 (2861.86–5733.67)**	**1.72% (1.54–1.89)**	**353463.17 (220266.54–538893.99)**	**122.96 (76.70–187.18)**	**1.68% (1.50–1.85)**
Australasia	46743.67 (27809.19–73799.01)	671.55 (400.88–1057.97)	0.67% (0.61–0.74)	487418.18 (328339.78–688305.63)	6805.30 (4579.69–9604.93)	1.11% (1.02–1.21)	14438.95 (8637.34–22510.45)	202.70 (121.22–316.19)	1.10% (1.00–1.19)
High-income Asia Pacific	245664.20 (148024.03–389455.98)	407.04 (245.68–644.58)	0.24% (0.21–0.27)	1858087.44 (1210770.99–2690103.93)	3035.79 (1974.21–4366.66)	0.47% (0.44–0.51)	55389.12 (32686.69–87186.05)	91.59 (54.00–143.69)	0.48% (0.45–0.51)
High-income North America	530916.80 (319634.87–832222.04)	598.57 (361.02–937.53)	1.94% (1.62–2.26)	5887893.07 (4200348.59–7926717.93)	6588.53 (4702.57–8859.18)	3.06% (2.64–3.49)	170714.47 (109674.79–253494.19)	191.70 (123.19–284.44)	2.98% (2.56–3.41)
Southern Latin America	49163.06 (29148.04–77228.99)	435.95 (258.65–684.87)	0.38% (0.33–0.43)	444548.30 (294197.61–629953.47)	3911.18 (2586.65–5538.83)	0.71% (0.66–0.77)	13238.00 (7856.31–20601.96)	116.78 (69.33–181.55)	0.69% (0.64–0.74)
Western Europe	351168.64 (209049.51–554627.23)	293.57 (175.15–463.39)	0.32% (0.27–0.37)	3359312.82 (2241220.21–4738454.54)	2736.44 (1822.37–3854.66)	0.68% (0.60–0.75)	99682.64 (59774.66–156201.12)	82.03 (49.12–128.51)	0.66% (0.59–0.74)
**Latin America and Caribbean**	**113618.04 (69200.69–174330.14)**	**147.08 (89.72–225.57)**	**0.85% (0.82–0.88)**	**628559.77 (404067.95–919053.87)**	**813.84 (523.30–1189.15)**	**0.89% (0.86–0.91)**	**18786.23 (10762.98–29881.33)**	**24.33 (13.95–38.68)**	**0.85% (0.82–0.88)**
Andean Latin America	13549.55 (8194.24–20798.56)	187.28 (113.28–287.47)	1.00% (0.95–1.05)	76263.63 (49232.40–111339.45)	1054.46 (680.35–1539.07)	1.1 % (1.05–1.16)	2294.97 (1236.89–3780.44)	31.76 (17.11–52.28)	1.08% (1.02–1.14)
Caribbean	11186.73 (6794.63–17217.87)	163.09 (99.23–250.55)	0.82% (0.79–0.84)	61404.82 (39566.88–89714.61)	895.95 (576.68–1307.65)	0.88% (0.85–0.90)	1837.35 (1031.15–2892.32)	26.91 (15.10–42.31)	0.82% (0.79–0.85)
Central Latin America	34154.26 (20631.22–52704.24)	110.40 (66.81–170.28)	0.91% (0.82–0.99)	191080.63 (121351.43–281406.29)	617.81 (392.56–909.56)	0.90% (0.82–0.98)	5789.43 (3265.40–9265.63)	18.72 (10.57–29.92)	0.86% (0.79–0.93)
Tropical Latin America	54727.51 (33379.09–83964.94)	169.70 (103.63–260.30)	0.85% (0.81–0.89)	299810.69 (193371.98–438251.88)	929.57 (599.85–1357.91)	0.89% (0.85–0.93)	8864.47 (5076.30–14148.37)	27.49 (15.75–43.85)	0.86% (0.82–0.9)
**North Africa and Middle East**	**393931.77 (240432.59–606559.19)**	**385.34 (235.48–594.35)**	**0.58% (0.55–0.60)**	**2309932.05 (1480826.90–3372765.51)**	**2278.21 (1462.70–3330.64)**	**0.66% (0.64–0.68)**	**68419.97 (39467.45–108660.30)**	**67.10 (38.78–106.46)**	**0.62% (0.60–0.64)**
**South Asia**	**1135067.41 (701991.98–1719980.77)**	**325.11 (200.88–494.17)**	**0.30 % (0.29–0.31)**	**6354306.60 (4097811.64–9253456.65)**	**1829.18 (1181.72–2664.30)**	**0.33% (0.32–0.34)**	**186580.3 (107091.59–295421.66)**	**53.40 (30.73–84.52)**	**0.34% (0.33–0.35)**
**Southeast Asia, East Asia, and Oceania**	**1832748.52 (1123154.23–2804353.39)**	**512.89 (314.69–784.02)**	**1.06% (0.95–1.17)**	**10891255.59 (7069349.40–15845656.20)**	**3055.81 (1983.69–4448.74)**	**1.19% (1.08–1.30)**	**327543.15 (190970.36–518118.07)**	**91.63 (53.43–145.07)**	**1.17% (1.06–1.28)**
East Asia	1492954.32 (915226.85–2290254.32)	535.90 (328.89–820.47)	1.16% (1.03–1.29)	8886271.30 (5768428.78–12936840.26)	3193.58 (2072.48–4652.53)	1.29% (1.16–1.42)	267315.89 (155868.52–421932.78)	95.88 (55.89–151.61)	1.27% (1.13–1.4)
Oceania	3744.64 (2281.56–5705.51)	485.01 (295.67–742.34)	0.27% (0.25–0.29)	22210.08 (14279.93–32056.11)	2915.25 (1881.33–4210.75)	0.33% (0.30–0.35)	663.14 (377.06–1060.72)	86.10 (49.07–137.02)	0.30% (0.28–0.32)
Southeast Asia	336049.56 (204977.43–511368.12)	429.77 (262.43–656.41)	0.65% (0.63–0.67)	1982774.21 (1283408.67–2876868.62)	2555.91 (1657.99–3712.52)	0.76% (0.73–0.78)	59564.12 (34249.82–95222.81)	76.26 (44.03–121.59)	0.75 (0.73–0.78)
**Sub-Saharan Africa**	**176826.09 (108946.68–269379.62)**	**348.07 (214.52–532.25)**	**0.15% (0.13–0.17)**	**1005271.70 (649055.55–1463499.68)**	**1998.53 (1292.72–2911.66)**	**0.20% (0.18–0.22)**	**29963.02 (17342.20–47272.8)**	**59.09 (34.35–93.04)**	**0.20% (0.18–0.22)**
Central Sub-Saharan Africa	18075.89 (11134.11–27822.78)	325.24 (200.35–503.03)	0.04% (–0.02–0.11)	102416.64 (65759.01–149730.18)	1874.40 (1206.58–2743.22)	0.03% (–0.03–0.10)	3046.05 (1692.49–4898.7)	55.08 (30.67–88.44)	0.06% (–0.01–0.13)
Eastern Sub-Saharan Africa	62642.06 (38670.79–95398.71)	352.16 (217.52–537.88)	0.18% (0.16–0.20)	353567.43 (227594.41–515212.18)	2008.52 (1295.83–2927.90)	0.22% (0.20–0.24)	10549.33 (6111.16–16738.97)	59.42 (34.54–94.07)	0.23% (0.21–0.25)
Southern Sub-Saharan Africa	27605.02 (16971.27–42060.28)	411.63 (253.06–629.66)	0.35% (0.31–0.39)	158812.26 (102569.08–229556.99)	2387.41 (1543.83–3454.73)	0.4% (0.36–0.43)	4677.63 (2731.66–7453.79)	69.89 (40.92–111.26)	0.35% (0.31–0.39)
Western Sub-Saharan Africa	68503.12 (42169.99–103796.93)	329.78 (202.93–501.93)	0.07% (0.01–0.14)	390475.38 (252578.35–569443.41)	1895.51 (1228.96–2765.70)	0.13% (0.07–0.2)	11690.02 (6775.54–18377.49)	56.32 (32.75–88.39)	0.15% (0.08–0.21)

SDI, Socio-Demographic Index; GBD, Global Burden of Disease, Injuries, and Risk Factors Study; EAPC, Estimated Annual Percentage Change; UIs, Uncertainty Intervals; CI, Confidence Interval; YLDs, Years Lived with Disability. Bold values indicate the GBD super regions.

### 3.2 Geographical distribution and temporal evolution

**Figure 3:**
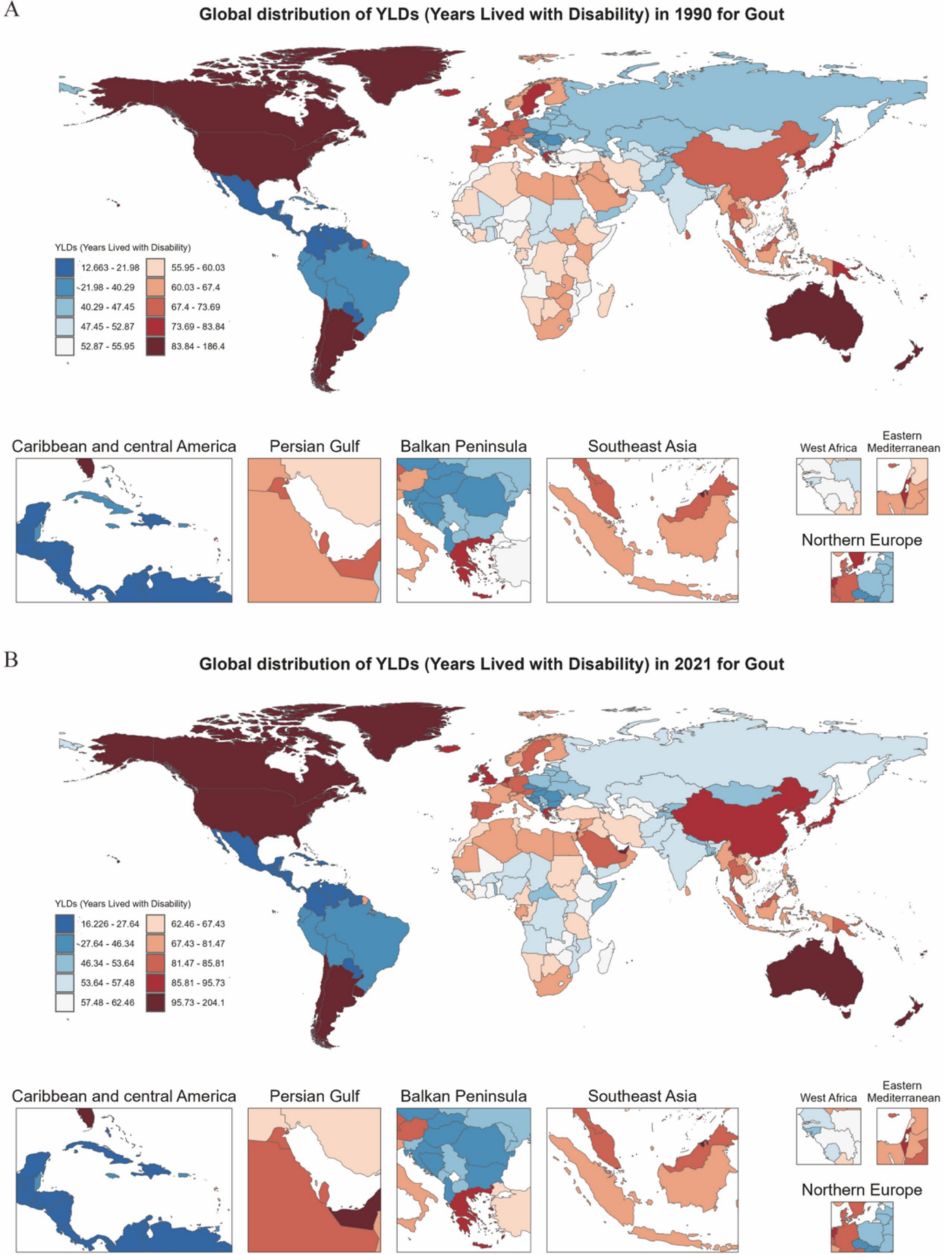
Heatmap of gout YLDs distribution among individuals aged 60 and above in 204 countries and regions globally for the years 1990 and 2021. **(A)** Distribution in 1990. **(B)** Distribution in 2021.

**Figure 4:**
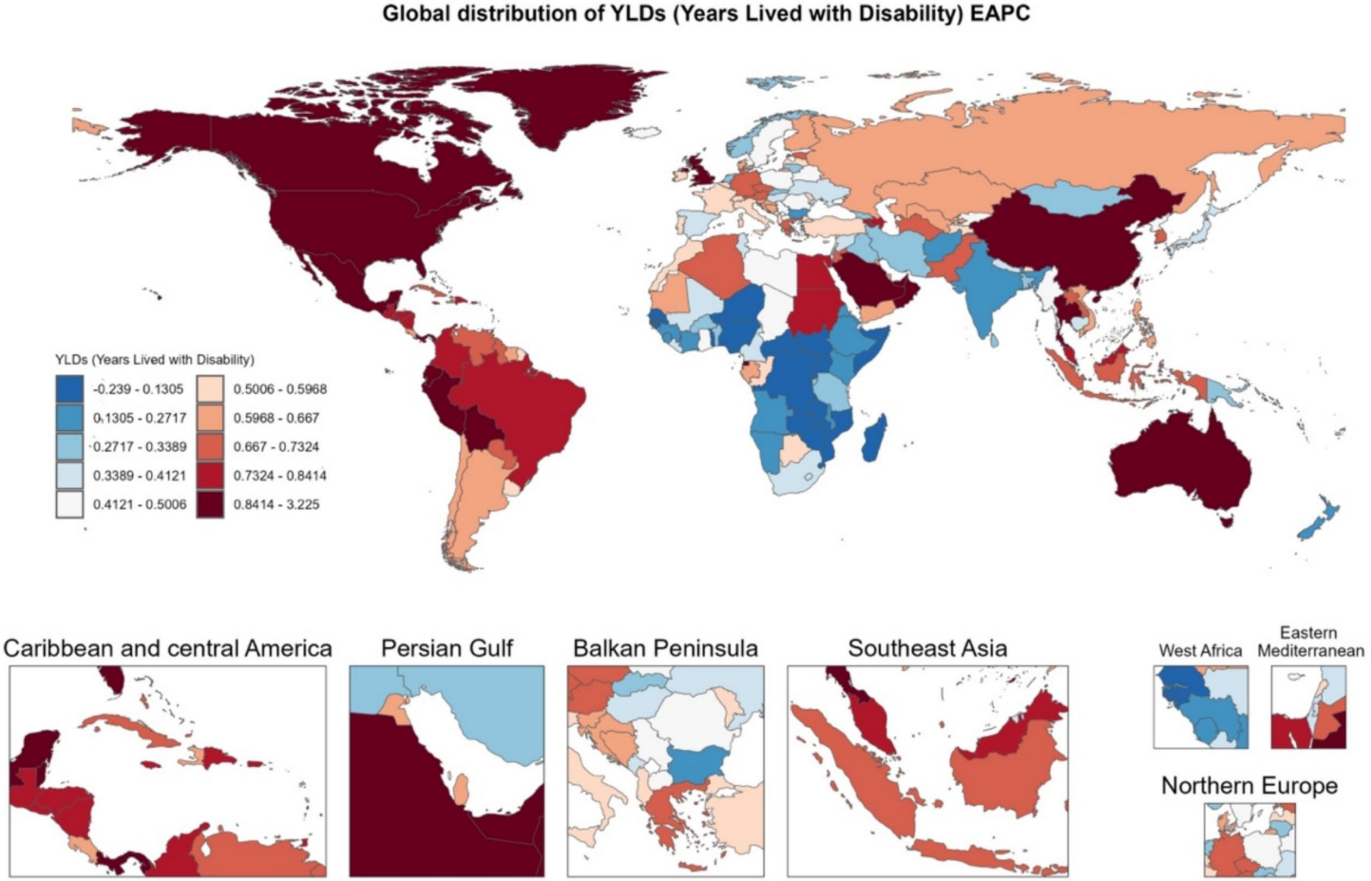
Heatmap of EAPC distribution of gout YLDs among individuals aged 60 and above in 204 countries and regions globally from 1990 to 2021.

Global YLDs heatmaps for 1990 and 2021 (Figure 3) show distinct geographical gradients. High-income regions (such as North America and Western Europe) already had high YLDs burdens in 1990, which further increased by 2021. Meanwhile, emerging economies (such as parts of South America, the Middle East, and East/Southeast Asia) transitioned from moderate to higher levels of burden. The Estimated Annual Percentage Change (EAPC) map (Figure 4) shows that North America, Australia, and some Gulf nations experienced the most significant increases in older adult gout YLDs, driven mainly by population aging and lifestyle changes. Supplementary Figure 1 illustrates the distribution of incidence and prevalence of gout among the older adults globally in 1990 and 2021. Notably, North and South America maintained high levels throughout the study period, while some Asian countries moved from moderate to higher levels. Supplementary Figure 2 further presents the EAPC for incidence and prevalence, indicating particularly steep increases in parts of the Americas and the Middle East, with even previously lower-burden areas (such as Sub-Saharan Africa) showing overall upward trends.

### 3.3 Socio-demographic differences and inequality

**Figure 5:**
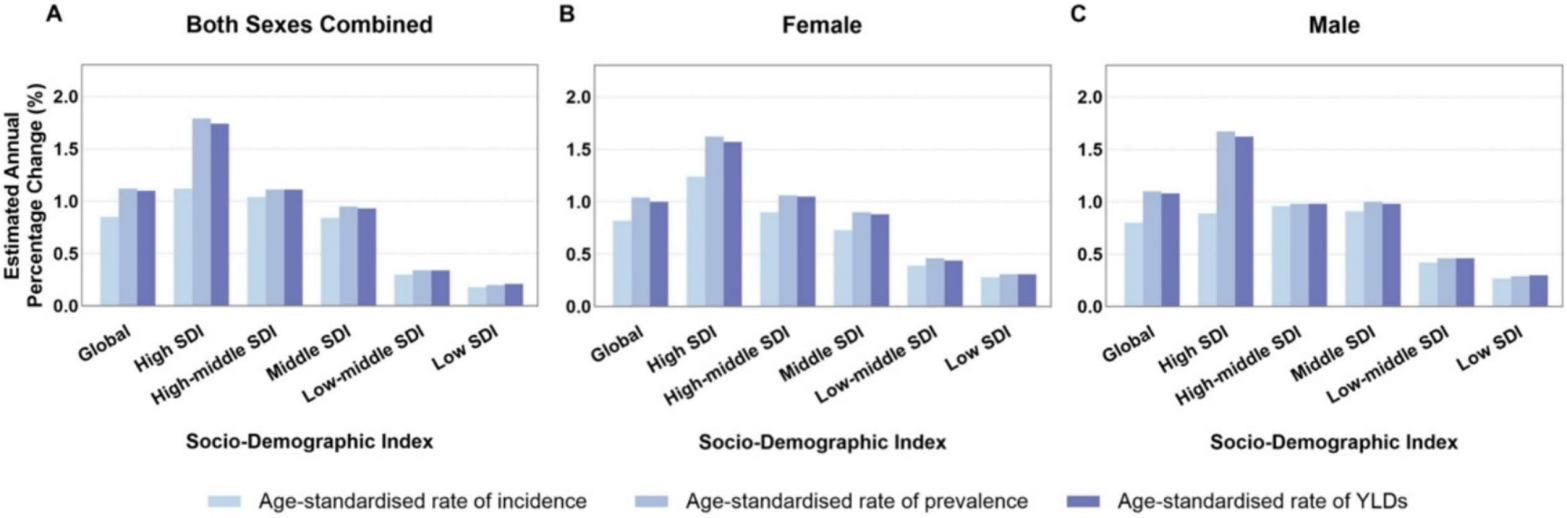
**(A–C)** Average annual percentage change in age-standardized rates of older adult gout globally and by SDI category from 1990 to 2021. SDI, socio-demographic index; YLDs, years lived with disability.

**Figure 6:**
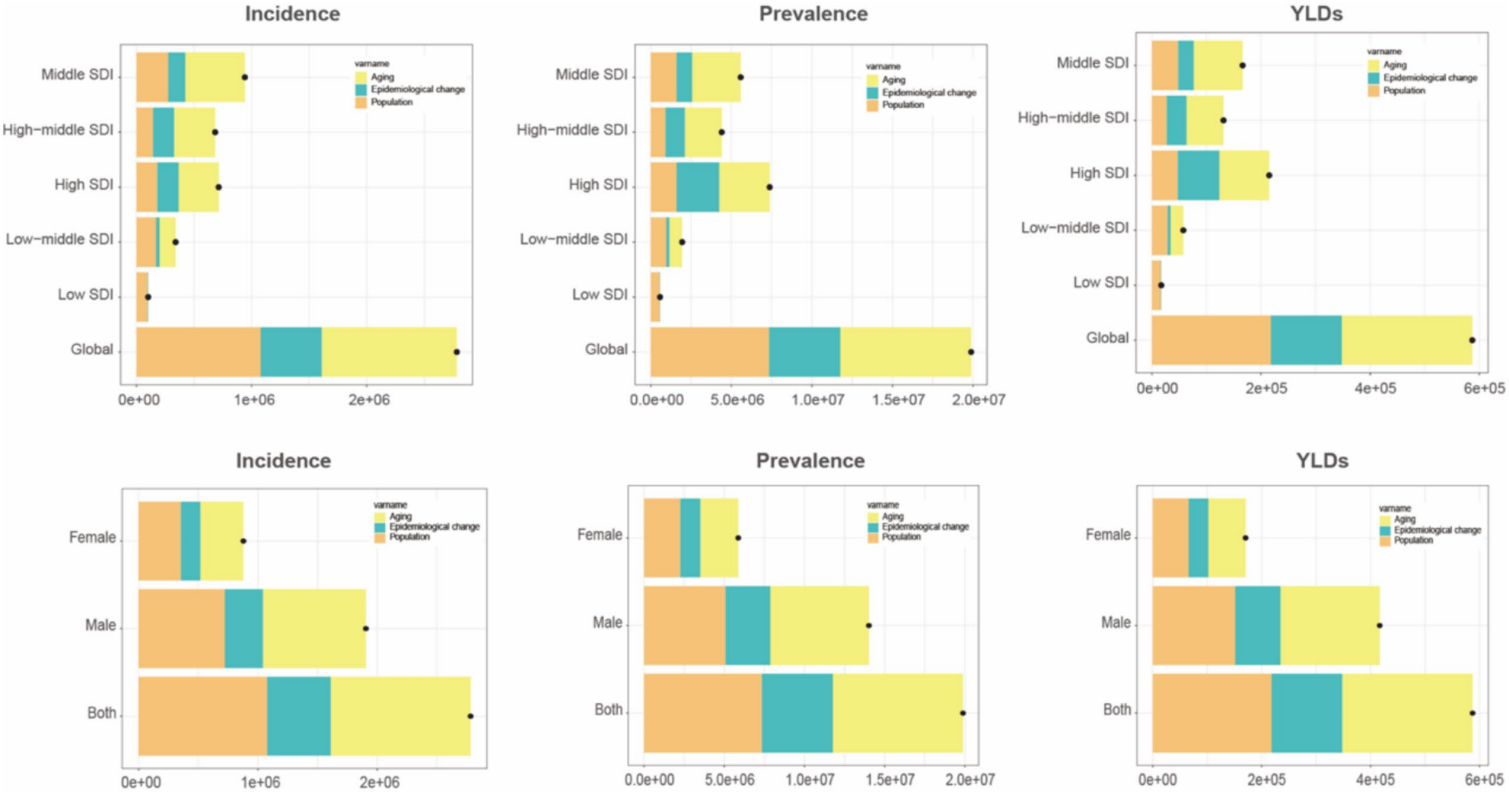
Trend decomposition analysis of incidence, prevalence, and YLDs for older adult gout from 1990 to 2021. YLDs, years lived with disability.

Figures 5A–C compare age-standardized rates (incidence, prevalence, and YLDs) across different Socio-demographic Index (SDI) categories from 1990 to 2021. High SDI and upper-middle SDI regions showed higher EAPC values, whereas low SDI regions experienced slower but still positive growth trends. Among genders, older women in high SDI regions saw more significant increases in prevalence and YLDs compared to men. Decomposition analysis (Figure 6) identified three primary drivers of increased gout burden among the older adults—population growth, population aging, and epidemiological changes (such as increases in hyperuricemia and metabolic risk factors). Population aging was the main driver in high SDI regions, while population growth had a greater impact in low SDI regions. Inequality indices (slope inequality index and concentration index) in Supplementary Figure 4 indicate that the gap in gout YLDs between high and low SDI countries widened from 1990 to 2021, with the burden increasingly concentrated in wealthier regions.

### 3.4 Future trend predictions (2022–2036)

Predictions using Bayesian Age-Period-Cohort (BAPC) models (Supplementary Figure 6) show that over the next 10–15 years, age-standardized incidence rates, prevalence, and YLDs for gout among older adults will remain unchanged or slowly decline. This suggests that the burden of gout among older adults may maintain 2020 levels or even decrease slightly.

## 4 Discussion

This study systematically analyzed the incidence, prevalence, and YLDs of gout in individuals aged ≥60 years using data from 204 countries and regions worldwide between 1990 and 2021, and employed the BAPC model to predict future trends. Results show that over the past three decades, the global burden of gout among older adults has continued to rise, with significant differences observed across genders, SDI groups, and geographic areas (3).

The findings reveal that during 1990–2021, the incidence, prevalence, and YLDs of gout among older adults (aged ≥60 years) have steadily increased, with most EAPCs being positive. Yearly increases in age-standardized metrics suggest this trend might be attributed to population aging. At the government level, on one hand, public welfare screening for blood uric acid levels among the older adult population can be implemented. On the other hand, further improvements can be made in the management of hypertension, chronic kidney disease, and other related conditions. Studies have shown that the ABCG2 gene encodes a high-capacity urate efflux transporter expressed in the human intestine and kidney, and it has been identified as a key genetic determinant in the pathogenesis of gout. It also plays a significant role in disease progression and severity. Therefore, with the increasing application of next-generation sequencing (NGS) in the healthcare field, it is now possible to precisely identify individuals at high risk for gout by targeting key susceptibility genes, paving the way for personalized diagnosis and treatment (18, 19). Additionally, promoting healthier lifestyles and diets can help mitigate the exacerbating effects of aging on gout (7, 20–22).

Geographical distribution analysis shows that North America, Australia, and some Middle Eastern countries have higher absolute levels and EAPCs of gout-related YLDs among the older adults, closely associated with their socioeconomic development levels, affluent lifestyles, and prevalent obesity and metabolic syndrome (23). Low-SDI regions may underestimate the true burden of gout due to limited healthcare resources, but this burden is expected to grow with urbanization and dietary pattern shifts. Frontier analysis further reveals significant differences in gout burdens among countries at similar SDI levels, which could be related to variations in screening coverage, health resource allocation, and the effectiveness of prevention and management within cultural contexts.

In this study, the BAPC model was also used to predict the burden of gout among older adults from 2022 to 2036, indicating that over the next 15 years, the incidence, prevalence, and YLDs of gout among older adults might remain stable or decrease.

Based on the GBD database, this study covers a wide range and uses standardized indicators for comparison. However, limitations exist: GBD data in some low-SDI regions may underestimate the burden of gout due to limited healthcare infrastructure; inconsistent diagnostic criteria may introduce potential errors; this study only explored the impact of high BMI on the epidemiology of gout without delving into the deeper effects of other comorbidities; Overall, the prevalence of gout and YLDs in the population aged 60 and above peaked around 2020, with the BAPC model’s predictions suggesting this year as a turning point. However, more data are needed to develop a more convincing predictive model. Future research should conduct longitudinal cohort studies in specific high-risk populations to investigate the coexistence of gout with other chronic diseases, the epidemiological characteristics of integrated interventions, and their economic benefits, while also strengthening research on gene–environment interactions.

## 5 Conclusion

Overall, our study indicates that gout in older adults is becoming an increasingly prominent public health issue, particularly evident in high-SDI countries. Predictive results suggest that the incidence, prevalence, and related disability burden of gout may decrease. Timely and coordinated responses to these challenges could further reduce the burden of gout on health systems and improve the quality of life for older adult populations globally.

## Data Availability

Publicly available datasets were analyzed in this study. This data can be found here: https://www.healthdata.org/research-analysis/gbd.

## Supplementary material

SUPPLEMENTARY FIGURE 1 Distribution of gout incidence and prevalence among individuals aged 60 and over in 204 countries and regions globally in 1990 **(A)** and 2021 **(B)**.

SUPPLEMENTARY FIGURE 2 Estimated annual percentage change (EAPC) in gout incidence and prevalence among individuals aged 60 and over globally from 1990 to 2021: Incidence **(A)** and Prevalence **(B)**.

SUPPLEMENTARY FIGURE 3 Relationship between gout-related disability-adjusted life years (DALYs) and SDI across countries in scatter plot **(A)** and concentration curve **(B)**.

SUPPLEMENTARY FIGURE 4 Inequality in gout YLDs among countries measured by Slope Index of Inequality **(A)** and Concentration Index **(B)** for the years 1990 and 2021.

SUPPLEMENTARY FIGURE 5 Frontier analysis of age-standardized gout burden by SDI from 1990 to 2020 **(A)** and trend classification between 1990 and 2020 **(B)**.

SUPPLEMENTARY FIGURE 6 Bayesian Age-Period-Cohort (BAPC) model predictions of gout burden among older adults: YLDs **(A)**, Prevalence **(B)**, and Incidence **(C)** from 1990 to 2036.
