# Supplementary figures and images for: Global burden and future projections of geriatric gout (1990–2021): a comprehensive analysis and Bayesian Age-Period-Cohort modeling
Source: Front Public Health. 2025 May 1;13:1577265. doi: 10.3389/fpubh.2025.1577265 (PMC12078167; doi:10.3389/fpubh.2025.1577265)

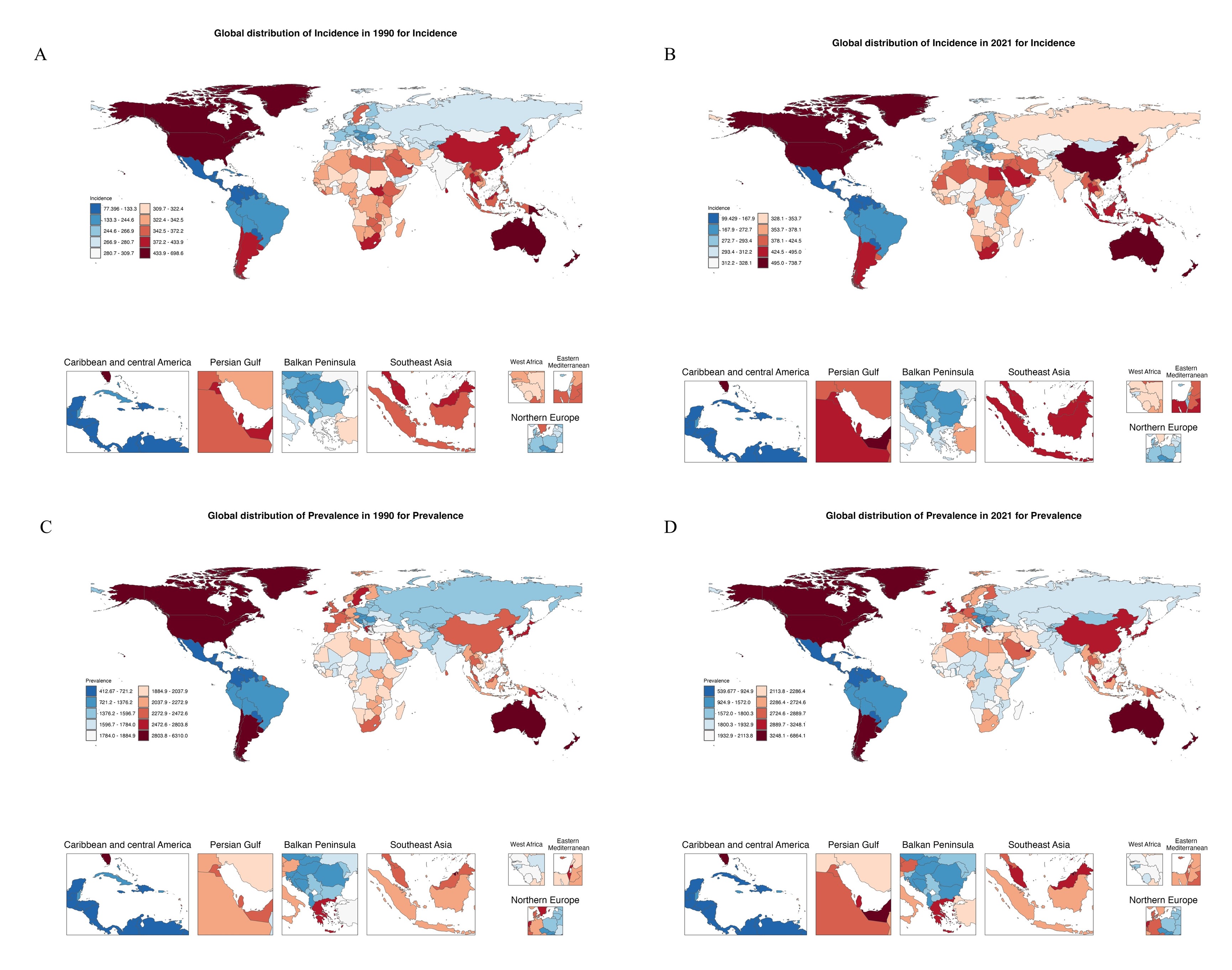

Supplement: SUPPLEMENTARY FIGURE 1 — Distribution of gout incidence and prevalence among individuals aged 60 and over in 204 countries and regions globally in 1990 (A) and 2021 (B). [file Image_1.JPEG]

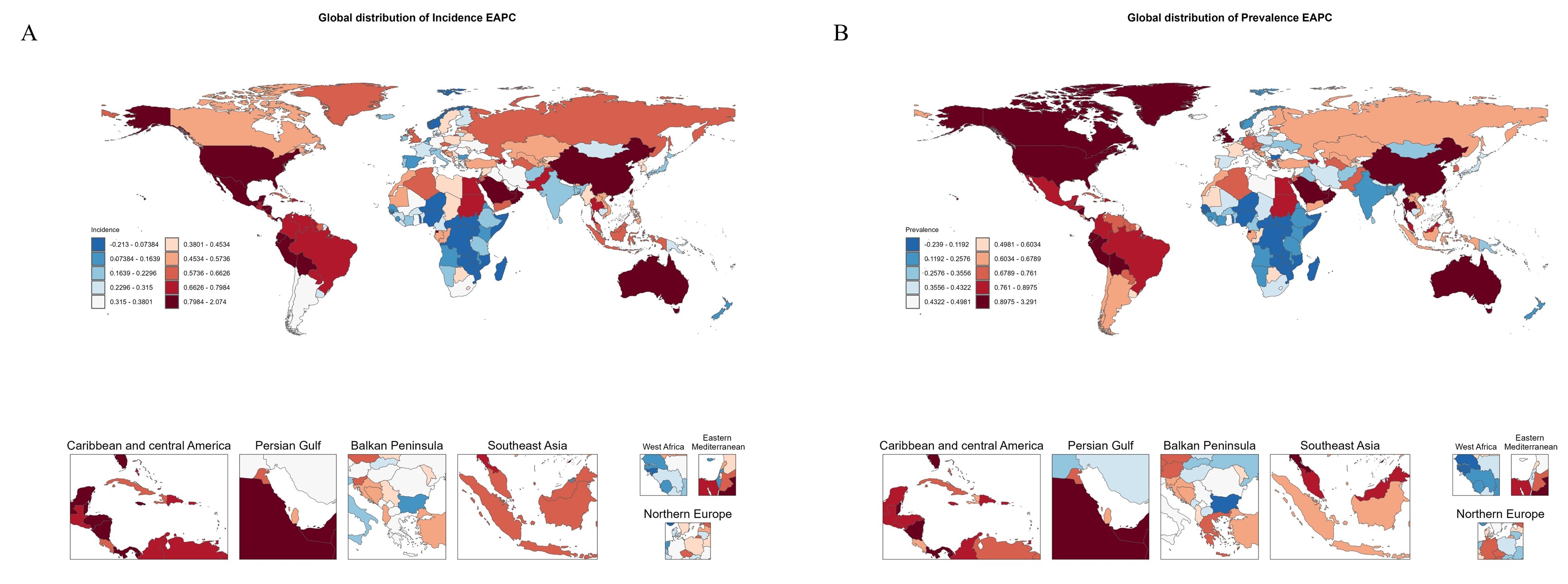

Supplement: SUPPLEMENTARY FIGURE 2 — Estimated annual percentage change (EAPC) in gout incidence and prevalence among individuals aged 60 and over globally from 1990 to 2021: Incidence (A) and Prevalence (B). [file Image_2.JPEG]

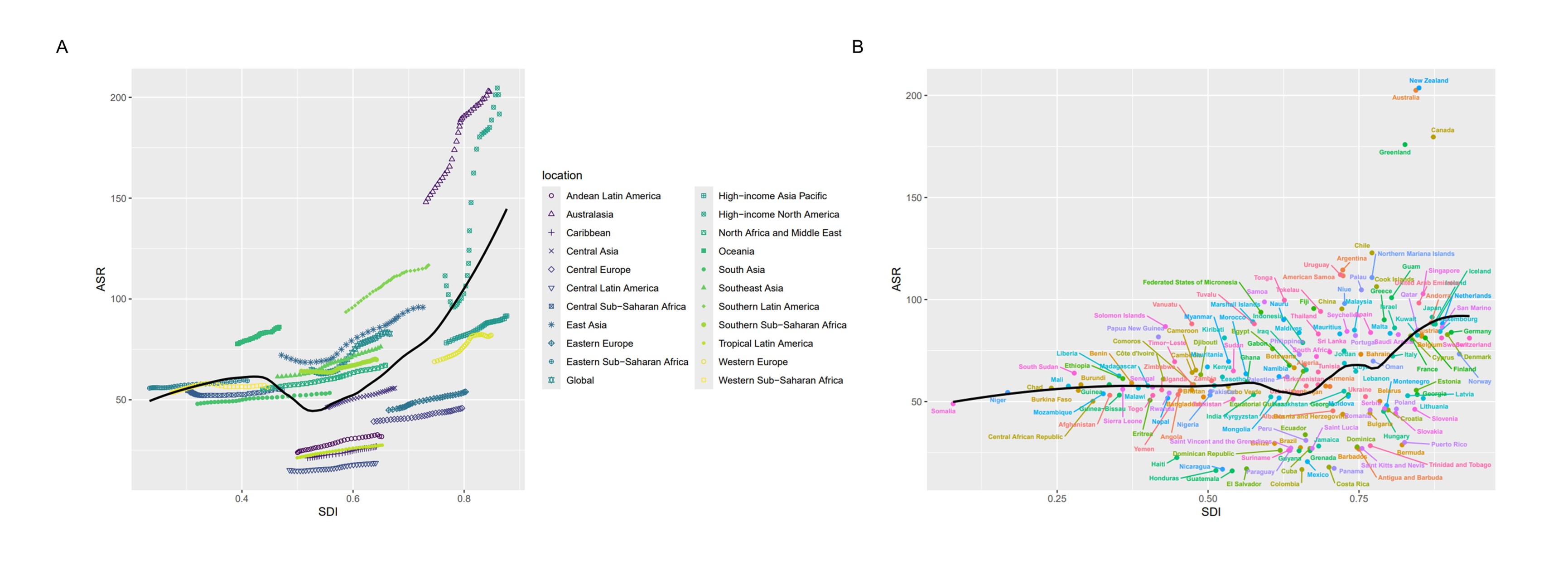

Supplement: SUPPLEMENTARY FIGURE 3 — Relationship between gout-related disability-adjusted life years (DALYs) and SDI across countries in scatter plot (A) and concentration curve (B). [file Image_3.JPEG]

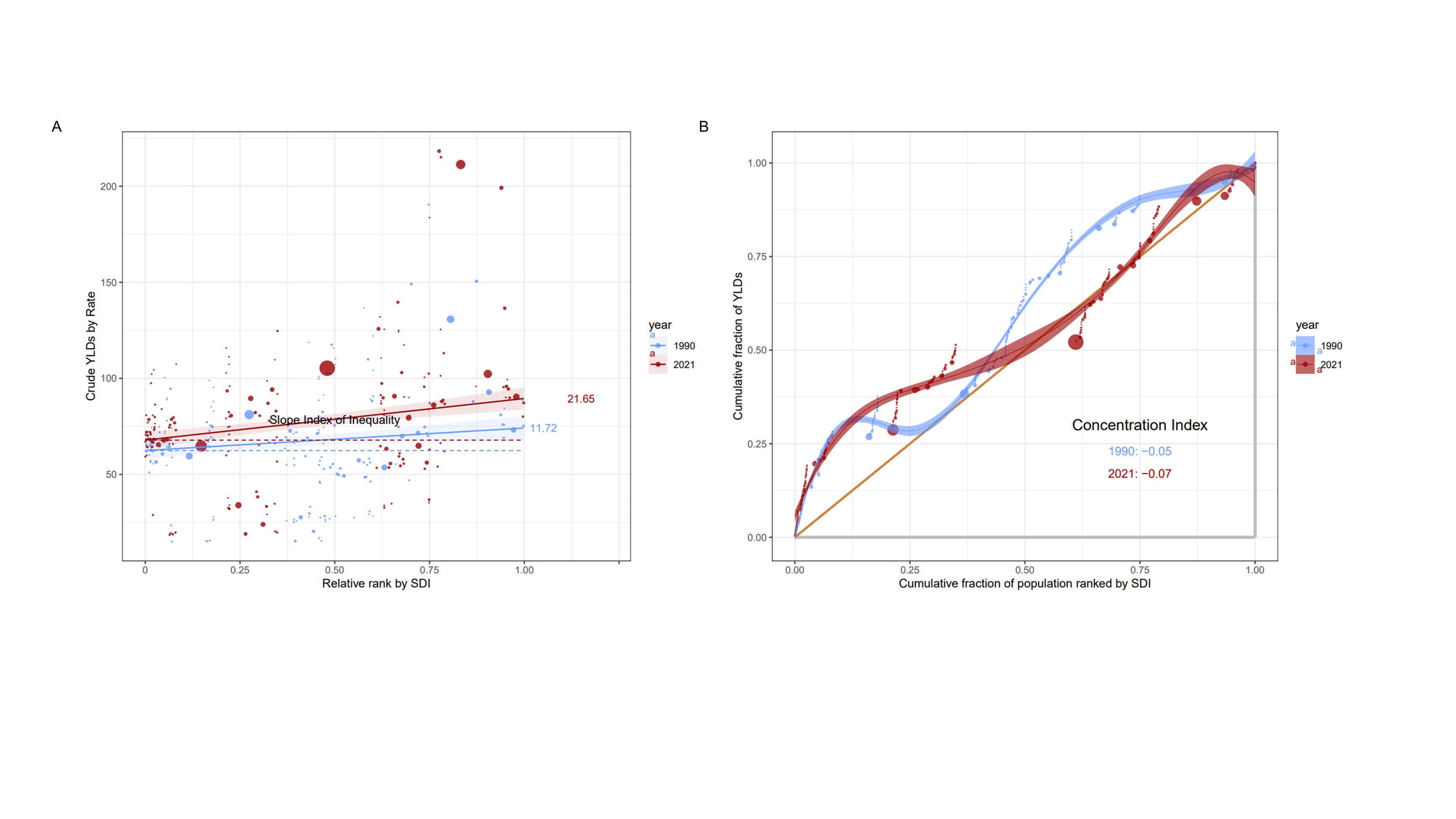

Supplement: SUPPLEMENTARY FIGURE 4 — Inequality in gout YLDs among countries measured by Slope Index of Inequality (A) and Concentration Index (B) for the years 1990 and 2021. [file Image_4.JPEG]

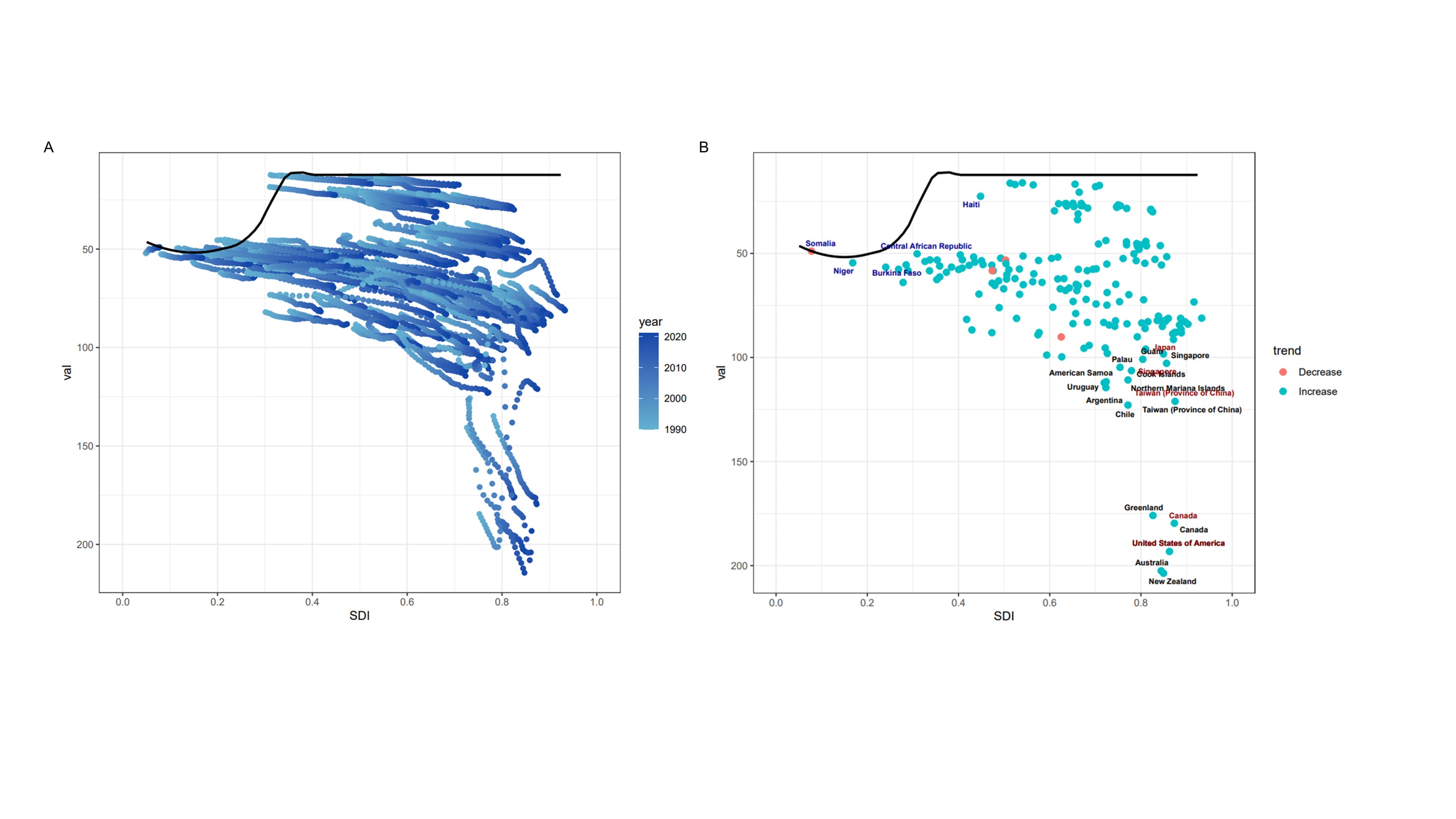

Supplement: SUPPLEMENTARY FIGURE 5 — Frontier analysis of age-standardized gout burden by SDI from 1990 to 2020 (A) and trend classification between 1990 and 2020 (B). [file Image_5.JPEG]

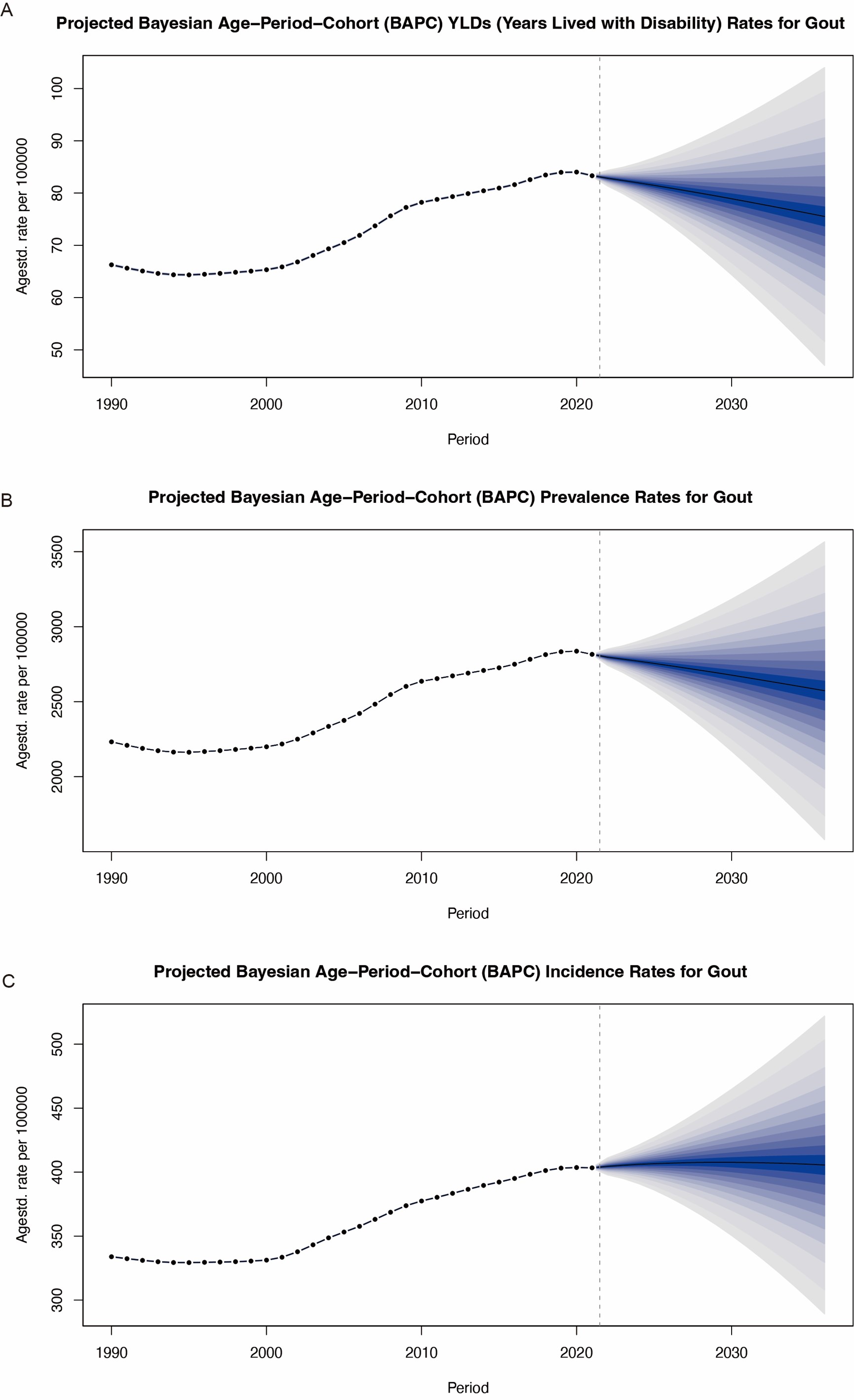

Supplement: SUPPLEMENTARY FIGURE 6 — Bayesian Age-Period-Cohort (BAPC) model predictions of gout burden among older adults: YLDs (A), Prevalence (B), and Incidence (C) from 1990 to 2036. [file Image_6.JPEG]
